# Successful Use of Aspirin, Apixaban, and Viscoelastography in a Patient with Severe COVID Disease and Allergy to Porcine Products

**DOI:** 10.1177/0018578721990898

**Published:** 2021-01-29

**Authors:** Jacob A. Reeder, Tessa R. Reynolds, Brian W. Gilbert

**Affiliations:** 1 Department of Pharmacy, Wesley Medical Center, Wichita, KS, USA

**Keywords:** anticoagulants, allergy, critical care

## Abstract

Coagulation abnormalities are frequently described in patients with severe acute respiratory syndrome coronavirus 2 (SARS-CoV-2). Common thromboprophylaxis and anticoagulation treatment strategies include the use of heparinoid therapy. We describe a 57-year-old woman with an allergy to porcine products that was started on apixaban for anticoagulation therapy given her allergy profile and need for venous thromboembolism prophylaxis. Apixaban and aspirin therapy were optimized with the support of serial viscoelastography and platelet function assays. Our patient experienced respiratory failure requiring intubation for 7 days but was successfully weaned to room air, tolerated a regular diet, and ultimately discharged to home after a 17-day hospital course. Here we report the safe and successful use of aspirin, apixaban, and viscoelastography for COVID-19-associated coagulopathy.

## Background

The World Health Organization has characterized the coronavirus disease 2019 (COVID-19) as a global pandemic in early March of 2020. The virus responsible for this disease has been defined as severe acute respiratory syndrome (SARS) coronavirus 2 (SARS-CoV-2). SARS-CoV-2 targets the respiratory epithelium, causing acute lung injury with potential for respiratory failure.^1^ While much is still unknown about the downstream complications of this disease, an initial coagulopathy has been characterized by increased level of clotting factors, enhanced platelet activation, and suppressed fibrinolyisis for those with severe illness.^1,2^ Exact definitions differ between consensus groups and societies, but the laboratory findings in COVID-19-associated coagulopathy can include: elevated d-dimer, elevated prothrombin time (PT) and international normalized ratio (INR), and increased platelet count and fibrinogen. Though these are surrogate markers, the abnormalities noted in these lab values have correlated with a significant increase in thromboembolic complications.^3^ Notably, Klok et al. reported an incidence of venous thromboembolism (VTE) in 27% of patients receiving standard thromboprophylaxis, while others have evaluated empiric full dose anticoagulation regardless of clot presence.^4^ The formation of VTE during COVID, even in patients receiving prophylactic or full doses of anticoagulation, have been described which has prompted many to initiate antiplatelet regimens in addition to anticoagulation. However, the addition of concomitant antiplatelet regimens with full dose anticoagulation may increase the patients risk for bleeding making monitoring of coagulopathy imperative. Thromboelastography (TEG) can measure whole blood clot formation and breakdown and is ideal for critically ill patients.^5^ Critically ill patients are already at an increased risk for VTE for numerous reasons, and COVID-19 potentiates that risk.^4,6^ The importance of recognizing this risk is imperative to mitigating morbidity and mortality in these patients.^3^

## Case

A 57-year-old woman presented to our emergency department with an 8-day history of cough, myalgia, dysgeusia, and endorsed increasing shortness of breath. Her past medical history included arthritis, type II diabetes mellitus, hyperlipidemia, hypertension, obesity, and coronary artery disease requiring stent placement. Of note, the patient’s allergy history included pork and porcine products. Upon initial evaluation, she was found to be febrile (100.4°F), tachycardic with a heart rate of 102 beats per minute, and an oxygen saturation of 54% on room air. Empiric antimicrobial treatment was started, and intravenous fluid administration was withheld for high suspicion of COVID-19 disease. Chest X-ray findings revealed diffuse increased interstitial and airspace opacities through the lungs. A nasopharyngeal swab resulted positive for SARS-CoV-2 via a polymerase chain reaction. Her initial baseline inflammatory labs included: C-reactive protein >190 mg/L, ferritin 306 ng/mL, lactate dehydrogenase 669 units/L, and a d-dimer 1087 ng/mL. Other significant findings included: white blood cell count 11 100/uL, red blood cell count 3.95/µL, hemoglobin 10.8 g/dL, hematocrit 34.1%, and platelet count of 397 000/uL.

Chemical venous thromboembolism prophylaxis was warranted; however, our patient had a significant allergy to pork and porcine products. Due to her allergy profile, heparinoids were avoided. The decision was made to start a direct oral anticoagulant (DOAC), apixaban 5 mg by mouth twice daily and resume her home regimen of aspirin 81 mg by mouth daily. Since the patient was started on full anticoagulation with apixaban, the decision was made to hold her clopidogrel due to the concern for increased bleeding risk. She was also started on 6 mg of dexamethasone by mouth once daily. Over the next 24 hours, the patient required increasing respiratory support to maintain oxygen saturation with bilevel positive airway pressure noninvasive ventilation. The following day, her d-dimer increased to 2287 ng/mL and a viscoelastic test (TEG^®^) was performed to assist with coagulopathy identification. We also wanted to ensure a therapeutic effect with apixaban given the significant drug-drug interaction with dexamethasone being a p-glycoprotein inducer. The initial TEG^®^ resulted with a shortened K time and increased maximum amplitude reflecting a hypercoagulable state. Given these objective findings and clinical course of the patient, the decision was made to increase the apixaban to 10 mg by mouth twice daily (BID). Overnight, the patient required intubation for refractory hypoxemia. Daily TEG^®^ labs were performed to assess the patient’s response and assist in pharmacologic management, including dose titration of apixaban and aspirin which are included in Table 1. On day 3, the TEG^®^ resulted with an increase in the maximum amplitude from 80 to 82 mm suggesting an increase in platelet activity or quantity. Concomitantly, an increasing d-dimer and thrombocytosis was also present. Therefore, the decision was made to increase her dose of aspirin from 81 to 325 mg daily, while maintaining the apixaban dose at 10 mg BID. A platelet function assay was drawn the next day to provide a more sensitive measure of platelet reactivity. Our patients assay resulted 450 aspirin reaction units (ARU). A result of <550 ARU signifies aspirin induced platelet dysfunction. After 7 days of apixaban 10 mg twice daily and a decreasing d-dimer, the dose was decreased to 5 mg twice daily. Our patient was extubated on day 9 of hospital stay and transferred out of the intensive care unit on day 11. She was successfully weaned to room air, tolerated a regular diet, and ultimately discharged to home after a 17-day hospital course.

**Table 1.** TEG and Inflammatory Marker Findings.

	Day 1	Day 2	Day 3	Day 4	Day 5	Day 6	Day 7
K Time (0.5-2.3 min)	—	0.8	1.0	0.8	0.8	0.8	0.8
Alpha angle (64°-80°)	—	79	77	82	86	82	79
Max amplitude (52-57 mm)	—	80	82	82	76	78	78
% Lysis 30 min (<7.5%)	—	1.4	0.8	0.3	0.2	2.3	0.9
C-reactive protein (<8.0 mg/L)	>190				50.2		
Ferritin (8-252 ng/mL)	306						
Lactate dehydrogenase (81-234 units/L)	669			615			
D-Dimer (<500 ng/mL)	1087	2287	7462	6036	7947	11 151	6256

TEG = thromboelastography.

## Discussion

Evidence for the most appropriate treatment in COVID-19 positive patients is constantly evolving. A guidance document for the management of this patient population is available through the National Institute of Health (NIH), however, nearly all the recommendations regarding antithrombotic therapy are based on expert opinion.^7^ Although some guidance is provided for COVID-19 positive patients transitioning from warfarin to a DOAC in the outpatient setting, limited direction has been provided when initiating DOACs for the use of confirmed or suspected VTE in the inpatient setting.^7^ The NIH document states, patients with COVID-19 who experience a thromboembolic event or who are highly suspected to have thromboembolic disease at a time when imaging is not possible, should be managed with therapeutic doses of anticoagulant therapy as per the standard of care for patients without COVID-19.^7^

In our patient, apixaban was chosen for chemical VTE prophylaxis based on allergy history limiting the use of heparinoid products. Additional agents that could be used in this setting include other DOACs, direct thrombin inhibitors, or fondaparinux. The initial dosing regimen of 5 mg twice daily was selected given the need for pharmacologic VTE prophylaxis and was only escalated to 10 mg twice daily once a high suspicion for thromboembolic disease was appreciated via TEG^®^. No radiographic imaging was performed to confirm the presence of thromboembolism. Our patient remained on aspirin monotherapy and apixaban 5 mg by mouth twice daily for 2 wk post discharge.

A study performed by Cui et al. reported a sensitivity of 85% and specificity of 88.5% for venous thromboembolism in COVID-19 positive patients with a d-dimer >1.5 mg/L.^8^ Although the d-dimer is a non-specific acute phase reactant, in the setting of a positive COVID-19 diagnosis, providers should consider utilizing a similar threshold to guide anticoagulation initiation or escalation. An additional tool that can be used to quantify the presence of coagulopathy is thromboelastography.^5^ Our facility utilizes the TEG^®^ 5000 Thromboelastograph^®^ Hemostasis Analyzer System. This allows clinicians to identify coagulopathy via whole blood rather than through individual conventional testing. With the help of TEG^®^, we were able to optimize pharmacologic therapy to treat our patients’ specific hypercoagulable profile.

The apixaban package insert (PI) describes avoiding concomitant use of p-glycoprotein inducers as this reduces drug levels in the blood.^9^ Medications included in the PI to avoid include carbamazepine, rifampin, and St Johns wort.^9^ While dexamethasone is not listed, the theoretical concern for a decrease in serum concentrations remains. An ongoing study in multiple myeloma patients is investigating this exact drug-drug interaction, and although this is a select patient population, in vivo data such as anti-Xa levels and plasma apixaban levels are being collected. Until further data are collected and published describing this drug-drug interaction, TEG^®^ can aid clinicians with quantifying the significance.

Although there remains a paucity of evidence for anticoagulation practices in severe COVID-19 disease, several randomized controlled trials are ongoing evaluating multiple thromboprophylaxis strategies. Moving forward, our group will continue to use thromboelastography as an adjunct to conventional coagulation parameters to guide anticoagulation decisions and consider apixaban as an appropriate alternative to heparinoids.

## Conclusion

This is the first case report to our knowledge highlighting the safe and successful use of aspirin and apixaban for COVID-19-associated coagulopathy in a patient with allergies to porcine containing products.

## References

[1] Connors JM Levy JH. COVID-19 and its implications for thrombosis and anticoagulation. Blood. 2020;135(23):2033-2040. doi:10.1182/blood.2020006000 32339221 PMC7273827

[2] Iba T Levy JH Levi M Connors JM Thachil J. Coagulopathy of coronavirus disease 2019. Crit Care Med. 2020;48(9):1358-1364. doi:10.1097/CCM.0000000000004458 32467443 PMC7255402

[3] Tang N Bai H Chen X Gong J Li D Sun Z. Anticoagulant treatment is associated with decreased mortality in severe coronavirus disease 2019 patients with coagulopathy. J Thromb Haemost. 2020;18(5):1094-1099. doi:10.1111/jth.14817 32220112 PMC9906401

[4] Klok FA Kruip MJHA van der Meer NJM , et al. Incidence of thrombotic complications in critically ill ICU patients with COVID-19. Thromb Res. 2020;191:145-147. 32291094 10.1016/j.thromres.2020.04.013 PMC7146714

[5] Gilbert BW Bissell BD Santiago RD Rech MA. Tracing the lines: a review of viscoelastography for emergency medicine clinicians. J Emerg Med. 2020;59(2):201-215. 32418869 10.1016/j.jemermed.2020.04.009

[6] Miri M Goharani R Sistanizad M. Deep vein thrombosis among intensive care unit patients; an epidemiologic study. Emerg (Tehran). 2017;5(1):e13. 28286820 PMC5325881

[7] COVID-19 Treatment Guidelines Panel. Coronavirus disease 2019 (COVID-19) treatment guidelines. National Institutes of Health. 2020. Updated 2020. Accessed August 23, 2020. https://www.covid19treatmentguidelines.nih.gov/34003615

[8] Cui S Chen S Li X Liu S Wang F. Prevalence of venous thromboembolism in patients with severe novel coronavirus pneumonia. J Thromb Haemost. 2020;18(6):1421-1424. 32271988 10.1111/jth.14830 PMC7262324

[9] Eliquis (apixaban) [package insert]. Princeton, NJ: Bristol-Myers Squibb Company; 2019.

